# Dynamics, Efficacies, and Adverse Effects of Maxillary Full-Arch Intrusion Using Temporary Anchorage Devices (Miniscrews): A Finite Element Analysis

**DOI:** 10.1155/2022/6706392

**Published:** 2022-10-07

**Authors:** Marzieh Mazhari, Mashallah Khanehmasjedi, Mohsen Mazhary, Nastaran Atashkar, Vahid Rakhshan

**Affiliations:** ^1^Department of Orthodontics, School of Dentistry, Ahvaz Jundishapur University of Medical Sciences, Ahvaz, Iran; ^2^Department of Civil and Environmental Engineering, ACECR Institute for Higher Education, Ahvaz, Iran; ^3^Department of Anatomy, Dental School, Azad University of Medical Sciences, Tehran, Iran

## Abstract

**Introduction:**

Absolute anchorages obtained from temporary anchorage devices (TADs, miniscrews) considerably facilitate dental movements and make some very difficult movements such as full-arch intrusions possible. Despite the significance of assessing strategies to fully intrude the arch using mini-implants, there is no study in this regard except a few case reports. Therefore, we simulated/tested 4 scenarios.

**Methods:**

Four maxilla models were created with different miniscrews/appliances: (1) two miniscrews were placed distal to laterals and one in the mid sagittal region. (2) Two mini-implants were inserted in mesial of canines and 2 others between bilateral first and second molars, plus another TAD in the midpalatal area, plus a transpalatal arch (TPA). (3) Two mini-implants were inserted between bilateral canines and first premolars and 2 others between bilateral first and second molars + TPA. (4) Two mini-implants were installed between lateral-and-canine and 2 miniscrews between second premolars and first molars + TPA. Intrusive forces (80 g anterior, 150 g posterior) were exerted using stainless-steel coil springs. Stresses/displacements were measured. Risk of external root resorption was evaluated.

**Results:**

The highest amounts of incisor/molar intrusion were seen in model 1. Model 2 had fewer intrusions, but its control over undesired movements was greater. Model 4 drastically reduced molar intrusion and considerably increased premolar intrusion. Overall amounts of intrusion were highest in the first 2 models, marking them as proper candidates for cases needing greater intrusion extents. Model 2 may be useful when miniscrew loosening/failure is a concern, while model 1 is recommended when fewer miniscrews are allowed. Overall, the highest and lowest root resorptions might occur in models 1 and 4, respectively.

**Conclusions:**

Each model showed certain efficacies/drawbacks and thus is recommended for a particular set of cases. Therefore, depending on the diagnosis and treatment plan, one or more of these scenarios might be desirable.

## 1. Introduction

Excessive gingival display, also known as “gummy smile,” is an esthetic concern among dental patients, because it is generally considered unpleasant and causes many patients to seek treatment for this problem [1]. A gummy smile, in which more than 3 to 4 mm of gingival tissue is exposed when smiling, causes an esthetic disharmony. Anatomical landmarks that play a role in creating a gingival smile include the maxilla, lips, gingival structures, and teeth [1]. To achieve a beautiful smile, all these anatomical structures must be in harmony with each other [1]. The various causes of gummy smile are altered passive eruption of teeth, dentoalveolar extrusion, vertical maxillary excess, short or hyperactive muscles of the upper lip, or a combination of them [2].

Altered passive eruption can be corrected with crown lengthening surgery, which can be achieved through gingivectomy or apically positioned flap. When the hyperactive upper lip is the main cause of gummy smile, surgical or nonsurgical methods (botulinum toxin injection) can be used for treatment [3].

However, gummy smiles caused by dentoalveolar and maxillary height etiologies are much more difficult to handle. In the past, dentoalveolar extrusion and increased maxillary height could only be corrected through orthognathic surgery, which is an invasive procedure [3]. However, with the advent of temporary skeletal anchorage devices (TADs), it has been reported that in some cases, gummy smiles caused by dentoalveolar extrusion and increased maxillary height can be corrected [3, 4]. Some case reports have shown that a miniscrew can achieve the same effect as maxillary impaction with Le Fort I surgery, and this way a gummy smile can be corrected with the full intrusion of the maxillary arch [5].

Dental intrusion is often an integral part of orthodontic treatment because it improves the sagittal and vertical relationships of the incisors, corrects the angle between the incisors and subsequently the gingival line, and restores the beauty of the smile [6]. Nikolai defines intrusion as a form of translational tooth movement that moves apically along the longitudinal axis of the tooth, while Burstone defines it as the apical movement of the geometric radicular center relative to the occlusal plane or a plane defined based on the long axis of the tooth [7–9].

Despite the significance of strategies to fully intrude the arch (and correct the gummy smile caused by dentoalveolar extrusion and vertical maxillary excess) using absolute anchorages provided by mini-implants, there is no study in this regard except a couple of case reports [3–5]. Therefore, we aimed to simulate, for the first time, four different strategies of full arch intrusion using TADs and study their dynamics, efficacies, and potential adverse effects (such as the risk of root resorption, indicated by an excessively high PDL hydrostatic pressure which can collapse the capillaries and impair blood flow [10, 11]).

## 2. Materials and Methods

This study was an experimental 4-phase in silico simulation. First, the models were created and then loaded.

### 2.1. Modeling in the Mimics and 3-Matic Programs

Models of the bones, teeth, and PDLs were modeled in Mimics 3D image processing program (Mimics Research 21; Materialise NV; Brussels, Belgium) and 3-Matic software (Materialise). For this purpose, CT scan slices with an interslice distance of 1 mm (NewTom VGi; Finland) were fed into Mimics. Segmentation tools were used to create masks for the teeth, bones, and PDLs. Afterwards, the Calculate 3D command was used to create a 3D model of these elements. Next, the export functions of these programs were used to create all these parts in the “.stl” format.

To design the mini-implants, the Helix and Revolution commands of the Solidworks program (version 2018, Dassault Systemes; Paris, France) were used. For designing the orthodontic wires and brackets, the ANSYS program (ANSYS Workbench 2021, ANSYS Inc., Canonsburg, Pennsylvania, USA) was used. Finally, the elements were assembled together in the ANSYS environment. The titanium miniscrews were self-drilling and conical square threaded.

### 2.2. Geometry Conversion in the Geomagic Program

The Geomagic program (3D Systems, Morrisville, North Carolina, United States) was used to convert the parts exported in the “.stl” format from the Mimics and 3-Matic programs (Materialise) into “parts” in the “.stp” format.

### 2.3. Analysis in the ANSYS Program

Following altering all components to the “.stp” format, they were opened by ANSYS Workbench 2021 (ANSYS Inc.) for simulations.

### 2.4. Simulation Models

All models designed in this study were a simulation of the full arch intrusion of maxillary teeth. The thickness of the periodontal ligament was assumed to be uniformly 0.25 mm, and the alveolar bone crest was constructed following the curvature of the cementoenamel junction (CEJ), 1 mm apical to the CEJ. Prescription brackets were designed based on the 0.022-inch-slot MBT system. The position of the brackets on the teeth was also based on this system. A stainless steel (SS) 0.019′ × 0.025′ archwire was crated as the archwire in all models. The arch form was designed as oval. The midpoints of the incisal edge of the tips of the buccal cusps and the apex of the roots were used as landmarks to assess the extent of displacement. The occlusal plane was defined by connecting the midpoint of the central incisal edge and the mesiobuccal cusp of the first molar. The teeth, alveolar bone, brackets, periodontal ligament, and archwires were constructed using fine tetrahedron solid elements, and all isoparametric and linear elastic objects were assumed to be homogeneous. Owing to the displacement of the dentition within the basal bone, the model was limited to the nasal floor of the alveolar bone in all directions. The connection between the miniscrew and the bone was defined as a tight tie in all models. The miniscrew movements were negligible and thus were not reported.

**Figure 1:**
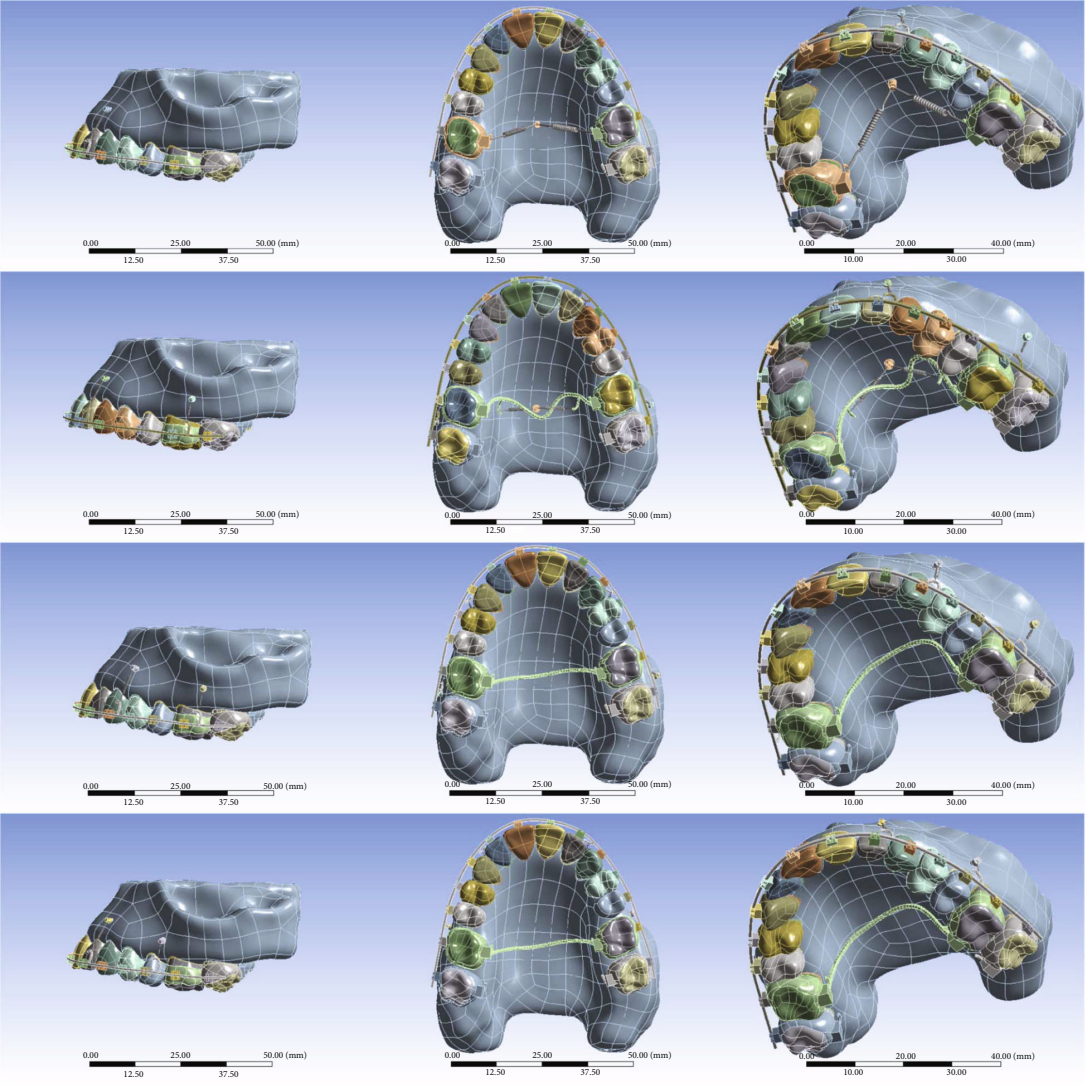
The models in use. From top to bottom: models 1, 2, 3, and 4.

Four different models were created with the above general descriptions and the following specifics (Figure 1). Two of them were inspired by two case reports [4, 12], but the other two were designed by the authors. In all models, the length of miniscrews were based on earlier references [4, 12, 13].

#### 2.4.1. Model 1

This model was partly derived from a case report [4]. Two miniscrews with a diameter of 1.6 mm and a length of 6 mm were placed distally to the lateral teeth along with closed stainless-steel (SS) coil springs for the intrusion of the maxillary anterior teeth. For the intrusion of the incisors, 80 grams of force was applied on each side. A palatal TAD (6 mm long, 1.8 mm in diameter) was placed in the mid sagittal region, parallel to the palatal root of the first molar. For molar intrusion, 150 g of force was applied on each side (Figure 1).

#### 2.4.2. Model 2

Four buccal miniscrews with a diameter of 1.6 and 6 mm were installed: two screws were placed in the mesial side of the canines and two screws between the first molar and the second molar. An 80 g force was exerted from each screw using SS closed coil springs. A mini-implant (with a diameter of 1.8 mm and a length of 5 mm) was inserted in the midpalatal; 80 grams of force was applied on each side of it with SS closed coil springs. A transpalatal arch (TPA) of 0.9′ wire was also placed (Figure 1).

#### 2.4.3. Model 3

This model was inspired by a case report [12]. In this model, two miniscrews were placed between the maxillary canines and the first premolars on each side, and two other miniscrews were placed between the first and second maxillary molars (1.6 mm in diameter and 6 mm in length) on each side. Intrusive forces of 80 g in the anterior region and 150 g in the posterior region were applied vertically to the maxillary archwire from the miniscrews through SS closed coil springs. A TPA made of 0.9′ wire was applied as well (Figure 1).

#### 2.4.4. Model 4

This model was rather similar to the 3rd model, apart from the locations of the mini-implants (1.6 mm in diameter and 6 mm in length): The anterior miniscrews were placed between the lateral and canine teeth on each side and between the second premolars and the first molars on each side. Intrusive forces of 80 g in the anterior region and 150 g in the posterior region were applied vertically using closed coil springs. The TPA in use was made of 0.9′ wire (Figure 1).

### 2.5. Material Properties

Materials in the models were assigned the properties explained in Table 1 [14–17]. The simulated spring type was SS closed-coil [18] with the following characteristics: wire diameter of 0.010 inch, lumen size of 0.030 inch, initial length range of 4-10 mm, and estimated stiffness of 0.67 N/Sq.mm.

### 2.6. Meshing

After applying the properties of the components, their meshing, which is one of the main parts of finite element analysis, was performed. To do this, the model was divided into smaller three-dimensional parts called elements, which were made up of a number of nodes. The total number of elements in the model was 133161 tetrahedral elements, and the number of nodes was 252999.

### 2.7. Boundary Conditions

**Figure 2:**
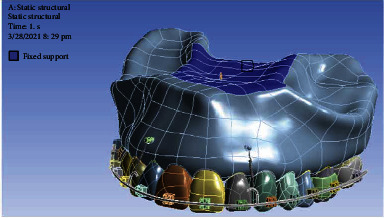
The fixed support of the maxilla.

In the next step, boundary conditions were applied: in this step, the fixed parts of the model were identified and forces were applied to the model. The maxilla was immobilized at its upper surface (Figure 2).

### 2.8. Outcomes

The duration for finite element simulations was 1 second. The created and loaded models were compared regarding hydrostatic stresses of PDLs, von Mises stresses, and displacements of all the components. Several methods can be used to explain tooth displacement, two of which are used in this study. Tooth movement can be described based on the displacement of each tooth and its bracket, meaning that an axis of local coordinates is drawn at the location of the bracket. Another way is to use an external reference such as a global coordinate system [19]. We used both of these systems to illustrate the movements of the teeth in the 3D space. The global axes were defined as follows: The *Y*-axis was the posterior-anterior axis with positive values indicating posterior movements and negative values indicating anterior movements. The *X*-axis was the lateral movement (right-left) axis, with positive values indicating the displacement towards the patient's left side, while negative values indicating the movement towards the patient's right side. The *Z*-axis was for the vertical movement, with positive values indicating intrusion (upward movement) and negative values indicating extrusion (downward movement). The local axes were defined individually for each tooth: The vertical axis was defined as exactly the global *Z* (vertical) axis. The mesiodistal axis was defined as the axis pointing from the distal (negative) to the mesial (positive) of each tooth. The buccolingual (or buccopalatal) axis was defined as the axis pointing from the buccal (negative) to the palatal (positive) of each tooth.

It is suggested that if the PDL hydrostatic pressure exceeds the capillary pressure in the area, the vessels will collapse and blood flow to that area will be impaired, increasing the risk of root resorption. Capillary pressure in the PDL is estimated to be about 0.002 to 0.005 MPa [11]. Therefore, compressive hydrostatic stresses at the PDLs were compared with -0.0047 MPa as a threshold for a significant increase of the risk of external root resorption [10, 11].

## 3. Results

### 3.1. Miniscrew Stresses

#### 3.1.1. Model 1

**Figure 3:**
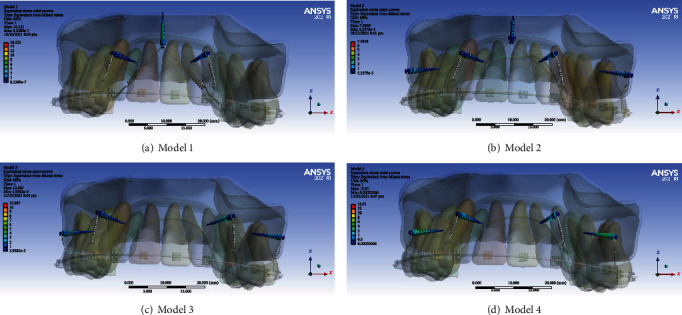
Mini-implant stresses (MPa). From top to bottom: models 1, 2, 3, and 4.

The palatal miniscrew endured more stresses than the two buccal miniscrews. The maximum stress in a great part of the buccal miniscrews was about 1 MPa. In the cervical and middle thirds of the buccal miniscrews, sections with a stress of up to 3 MPa were also seen. The minimum stress (up to 1 MPa) was seen in the head of the palatal miniscrew; the stress increased in the neck of the miniscrew and reached a maximum of 3 MPa. In the cervical and middle thirds of the threads, an increased stress and an approximate stress of 4-8 MPa was observed. In the apical part, like the head of the miniscrew, a little stress (up to 1 MPa) was seen (Figure 3, Table 2).

#### 3.1.2. Model 2

In the second model, the stress distribution on the buccal and palatal miniscrews was relatively similar. In most parts of the palatal miniscrew (such as the head, neck, and apex), the maximum stress on the miniscrew was 1 MPa, but in some parts of the threads, the stress was 1-2 MPa. In parts of the buccal miniscrew neck, stress was seen up to a maximum of 2 MPa. Stresses of approximately 2-4 MPa were observed in parts of the threads of the cervical third of the buccal miniscrews (Figure 3, Table 2).

#### 3.1.3. Model 3

In the third model, similar stresses were seen in the buccal miniscrews. In most of the neck of the miniscrews and their cervical half, a stress of 2-6 MPa was observed. At the head of the miniscrews and their apical parts, the maximum stress was 1 megapascal (Figure 3, Table 2).

#### 3.1.4. Model 4

In the fourth model, the stress levels in the miniscrews were relatively similar. Stress was minimal in the head and apical parts of the miniscrews (maximum: 0.5 MPa). The stress on the neck of the miniscrew increased to 1-2 MPa. In most of the threads of the cervical half of the miniscrews, a stress of about 1-4 MPa was observed. In a small fraction of the cervical third of each miniscrew, an approximate stress of 4-6 MPa was observed (Figure 3, Table 2).

Palatal mini screw in model 1 tolerated the greatest stress. Buccal miniscrews in model 2 were less stressed than other models (Figure 3, Table 2).

### 3.2. PDL Hydrostatic Pressure

#### 3.2.1. Model 1

**Figure 4:**
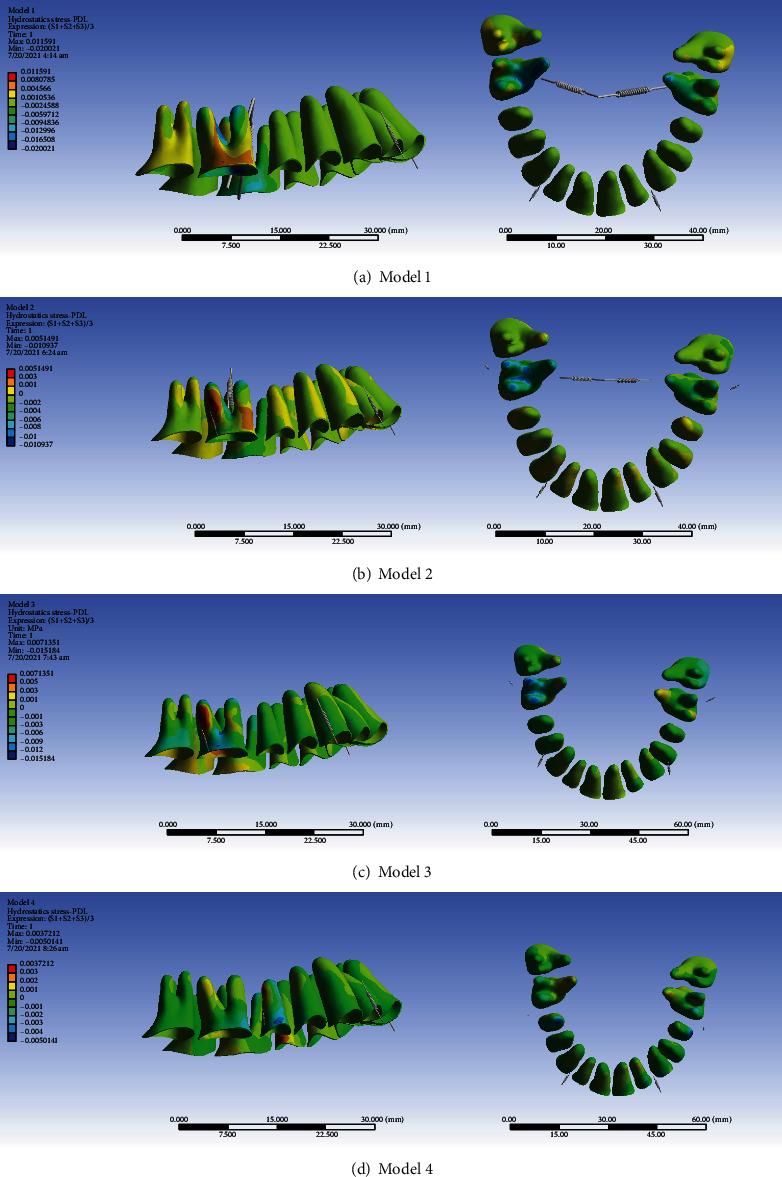
PDL hydrostatic stresses (MPa) from the lateral and occlusal views. From top to bottom: models 1, 2, 3, and 4. Positive values indicate tensile stresses while negative values show compressive pressures. Negative values below -0.0047 MPa (i.e., compressive pressures above 0.0047 MPa) pose a considerably higher external root resorption risk.

In the anterior teeth and premolars and parts of the second molars such as the palatal root and the palatal part and most of the buccal parts of the buccal roots, periodontal ligament compression was observed at a maximum of 0.002 MPa. The maximum tension was 0.004 which was observed in parts of the second molars. In the cervical and middle parts of the buccal roots of the first molar teeth, tensions up to 0.008 MPa were observed. In the apical parts of the buccal roots, the furca region, and the palatal roots of the first molars, compression zones of about 0.012 MPa were observed. The maximum compressive stress was 0.020 MPa, which was seen in the apical parts of the palatal roots of the first molars. The areas with a high risk of root resorption were the apical parts of the palatal root of the first molars (Figure 4, Table 3).

#### 3.2.2. Model 2

In most of the periodontal ligament of the anterior teeth and second premolars and molars, especially in the apical region and labial surfaces, mostly compression areas were observed. The maximum compressive hydrostatic pressure was 0.002 MPa and the maximum tensile hydrostatic stress was 0.001 MPa. In the mesiobuccal section of the mesiobuccal root of the first molars and the distal root of its distobuccal root, tension with a maximum value of 0.005 MPa was observed. Compression was seen in the cervicobuccal, furca and palatal roots of the first molars. The maximum compression extent was 0.0109 MPa. The cervicobuccal area, the furca, and the palatal root of the first molars were prone to external root resorption (Figure 4, Table 3).

#### 3.2.3. Model 3

Tensile stresses were seen in parts of the periodontal ligament of the central and lateral teeth, and compressive pressures were seen in other parts of the anterior teeth and premolars. The maximum compression was 0.001 MPa, and the maximum tension extent was 0.001 MPa. Tensile stresses were observed in small parts of the second molar with a maximum of 0.001 MPa. But in the major parts of the periodontal ligament of this tooth, compressive pressures were seen. Maximum compression was observed in the cervicobuccal area which was about 0.006. Tensile stresses were observed in small parts of the mesiobuccal root of the first molar and parts of its distobuccal root, the maximum of which was 0.007 MPa. Compression areas were seen in the cervicobuccal, furca, and roots of the first molar. The maximum compressive pressure in the apical parts of the buccal roots was about 0.0152 MPa (Figure 4, Table 3).

#### 3.2.4. Model 4

Tensile stresses were seen on the labial surfaces of the periodontal ligament of the incisors and distoapical sides of the premolars, and compression was seen in parts of the periodontal ligament of the anterior and first premolars and second molars. The maximum compression was 0.001 MPa, and the maximum traction was 0.001 MPa. In a small part of the second premolar periodontal ligament, tensile stresses were observed with a maximum of 0.0038 MPa. Compression of the second premolar periodontal ligament was observed in the cervicobuccal and apical parts, with a maximum value of 0.00501 MPa. In the first molar, tensile stresses were seen in parts of the distal and palatal roots. The cervicobuccal area of the second premolars and the apical areas of the buccal roots of the first molars were prone to external root resorption (Figure 4, Table 3).

The average stress of all models was negative and compressive. Model 1 had the greatest average (compressive) hydrostatic stress, while model 4 had the lowest average (compressive) stress. In model 3, the average stress was higher than that in model 2. The cervicobuccal and apical areas of the second premolars and the apical and cervicobuccal areas of the buccal roots of first molars were prone to external root resorption (Figure 4, Table 3).

### 3.3. Directional Displacements in the Global *Y*-Axis (Anterior-Posterior)

#### 3.3.1. Model 1

**Figure 5:**
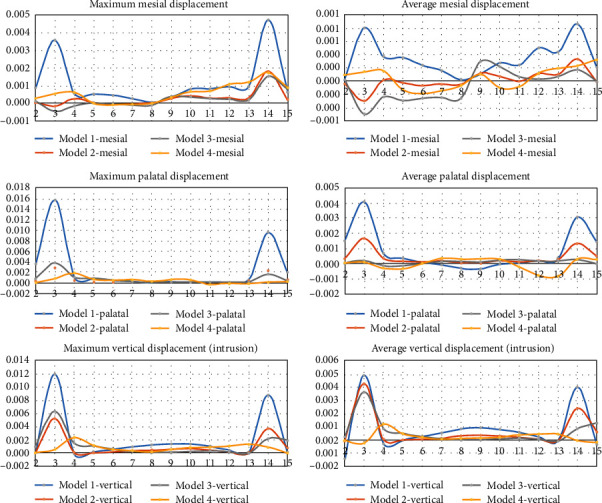
Maximum and average displacements of each of the 14 assessed teeth (mm), in each of the 4 models, in the local directions of mesial, distal, buccal, lingual, intrusive, and extrusive. The tooth numbers 2 to 15 represent the right second molar (#2) to the left second molar (#15), according to the *Universal Dental Notation* system (i.e., the molars are on the sides and the anterior teeth are in the center (the right central = #7, the left central = #8).

**Figure 6:**
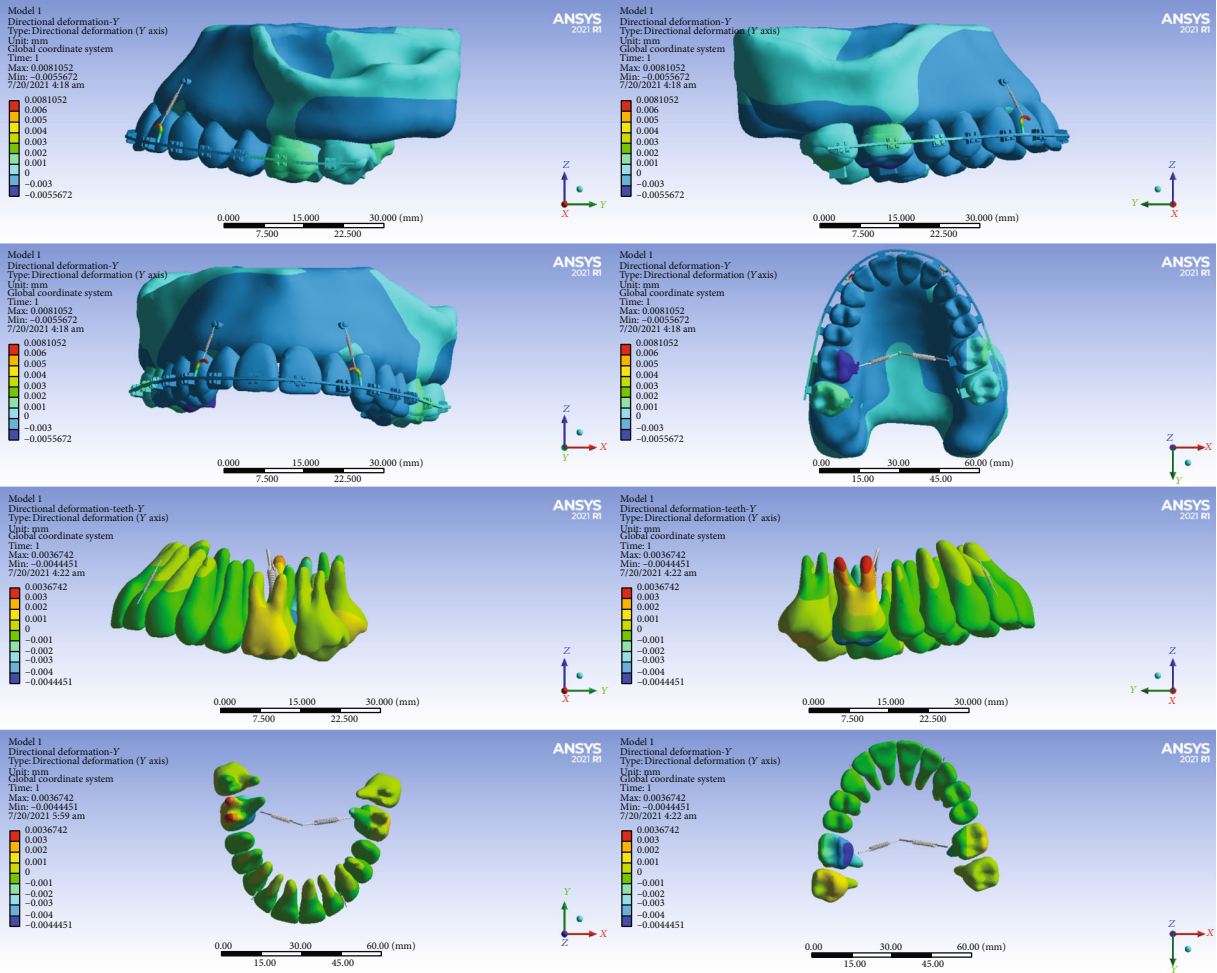
Displacements in the *Y* global direction (anterior-posterior) in model 1. Negative values indicate anterior movement, while positive values indicate posterior movement.

The crowns of the anterior teeth were displaced anteriorly (buccalized) for -0.001 mm, and their roots were displaced posteriorly (palatalized, up to 0.001 mm). The crowns of the premolars were mesialized. Most of their roots were also mesialized, but some apical parts of the roots in the first premolars were distalized. The crown and roots of the second molars were displaced posteriorly (distalized, up to 0.001 mm). The palatal cusps of the first molar teeth were displaced anteriorly (mesially) by a maximum of -0.0044 mm. The buccal part of the first molars moved posteriorly (were distalized) up to 0.002 mm. The buccal roots of the first molar teeth were displaced posteriorly (distalized, up to 0.0036 mm), and the palatal roots were moved anteriorly (mesialized, up to -0.003 mm) (Figures 5 and 6, Table 4).

#### 3.3.2. Model 2

**Figure 7:**
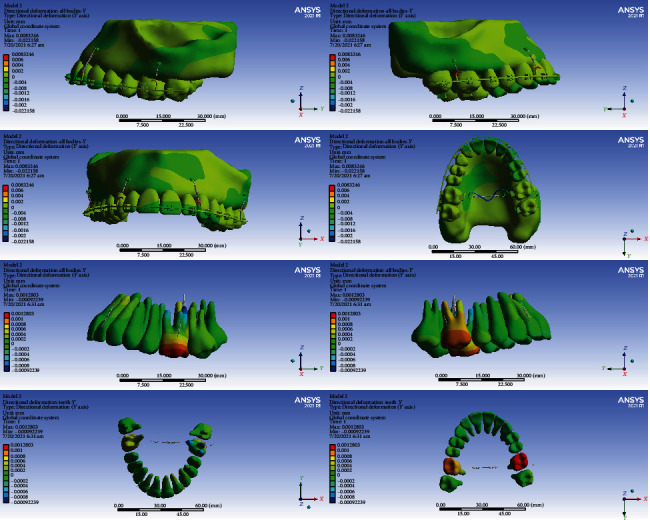
Displacements in the *Y* global direction (anterior-posterior) in model 2. Posterior movements are positive, while anterior movements are negative.

The crowns of the first molar teeth moved posteriorly (distally) and their roots anteriorly (mesially). The maximum displacement was observed in the crown of the first molars (0.0012 mm). The roots of the right first molar were distalized, and the cervical and middle parts of the left first molar were distalized and the apical parts were mesialized. The crowns of the anterior teeth were palatalized for up to 0.0002 mm, and their roots were palatalized for 0.0002 to 0.0006 mm. The second molars were distalized up to 0.0004 mm (Figures 5 and 7, Table 4).

#### 3.3.3. Model 3

**Figure 8:**
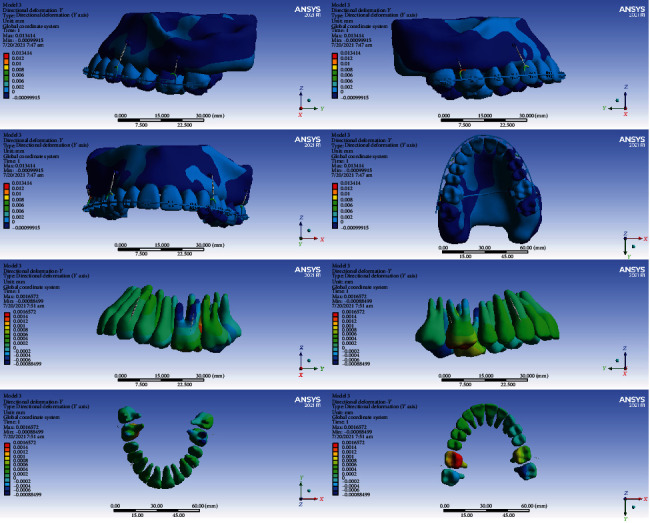
Displacements in the *Y* global direction (anterior-posterior) in model 3. Posterior and anterior displacements are positive and negative, respectively.

The anterior teeth and premolars moved slightly to the posterior (palatalized up to 0.004 mm). The premolars were distalized (maximum 0.004 mm). The greatest displacements were seen in the first molars. The roots of the first molars became mesialized (maximum root displacement: -0.0008 mm), and their crowns, especially the palatal cusps, became distalized (maximum crown displacement: 0.0016 mm). The crowns of the second molars were mesialized, and their roots were distalized (Figures 5 and 8, Table 4).

#### 3.3.4. Model 4

**Figure 9:**
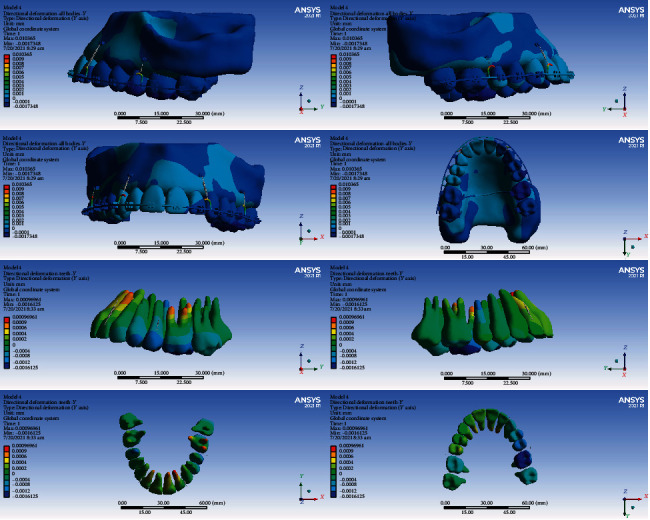
Displacements in the Y global direction (anterior-posterior) in model 4. The displacement to the posterior direction is positive, while anterior movements are negative.

The anterior teeth became palatalized: most displacements were at the root apex, and slight displacements were seen at the incisal edge. The highest amount of root palatalization was seen in the apical part of the lateral (up to 0.0009 mm). The crowns of the posterior teeth were mesialized, and their roots became distalized. The greatest displacements were observed in the first molar and premolars (up to 0.0016 mm) (Figures 5 and 9, Table 4).

In the anterior-posterior dimension, the second model and then the third model had the most displacements. The least displacement in this dimension was seen in the fourth model. The differences in displacements of the models 1 and 4 were very small (Figures 6–9, Table 4).

### 3.4. Displacements on the Global *X*-Axis (Left-Right)

#### 3.4.1. Model 1

**Figure 10:**
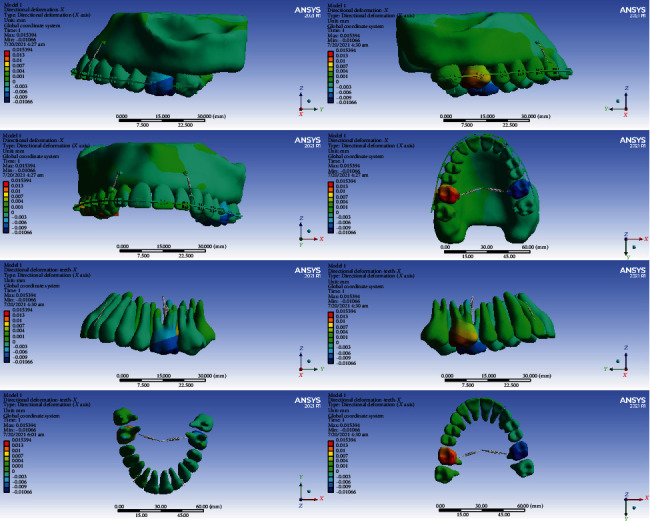
Displacements in the X global direction (left-right) in model 1. Positive values indicate movements to the patient's right side, while negative values indicate movements to the patient's left side.

The anterior teeth were mesialized (up to 0.003 mm). The premolars were palatalized (up to 0.003 mm). The crowns of the first molars became palatalized (up to 0.015394 mm), while the apex of their roots became buccalized (up to 0.003 mm) (Figures 5 and 10, Table 5).

#### 3.4.2. Model 2

**Figure 11:**
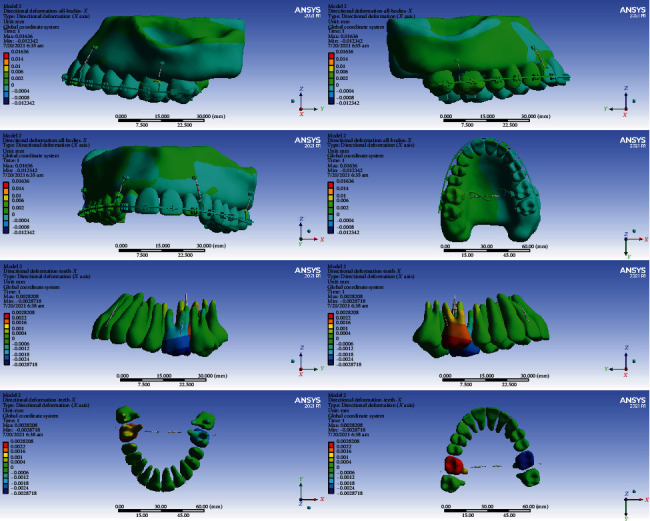
Displacements in the X global direction (left-right) in model 2. Positive and negative values indicate the movement towards the patient's right and left sides.

The posterior teeth became palatalized. In the first molar teeth, the rate of palatalization was the greatest (0.002 mm). The anterior teeth were mesialized up to 0.006 mm (Figures 5 and 11, Table 5).

#### 3.4.3. Model 3

**Figure 12:**
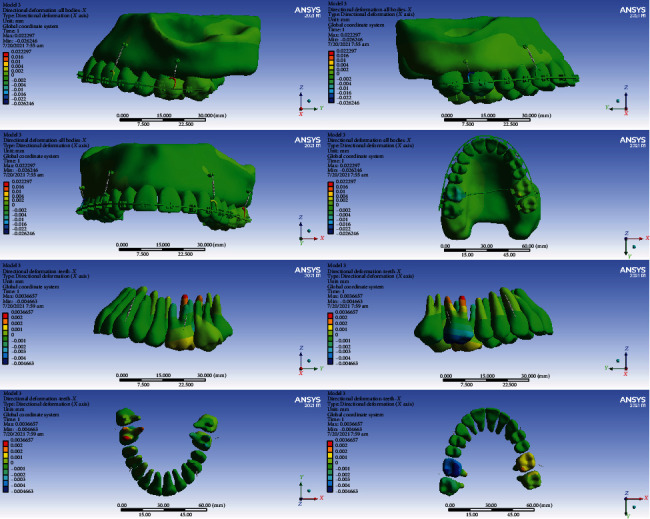
Displacements in the *X* global direction (left-right) in model 3. Positive values indicate movements to the patient's right side, while negative values indicate movements to the left side.

The crowns and roots of the left anterior teeth became mesialized. The crown and root of the right central moved distally. The crown and the cervical and middle parts of the right lateral moved distally, while the apical part moved mesially. The right canine crown was distalized while its root was mesialized. The crowns and roots of the left premolars were palatalized up to 0.001 mm. The crown of the right premolars moved buccally, and their roots moved palatally. The greatest movement was observed in the first molars (maximum displacement: 0.0046 mm). The crowns of the first molars were buccalized up to 0.0046 mm. Their roots became palatalized up to 0.0036 mm. The crowns of the second molars moved towards buccal, and their roots moved palatally (Figures 5 and 12, Table 5).

#### 3.4.4. Model 4

**Figure 13:**
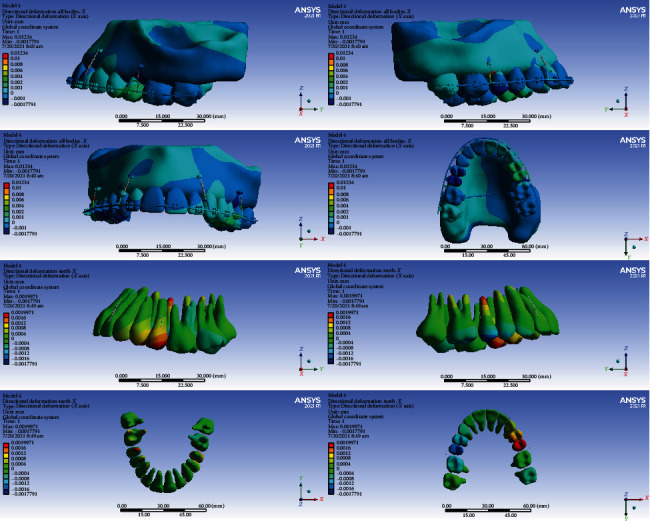
Displacements in the *X* global direction (left-right) in model 4. Positive values indicate movements to the patient's right side, while negative values indicate movements to the left side.

The left central and lateral were mesialized, while the other anterior teeth became distalized. The posterior teeth became buccalized. The displacement rate in most teeth was up to 0.0004 mm. The most extent of movement was observed in the second premolars at 0.00199 mm (Figures 5 and 13, Table 5).

In the X global axis, the highest displacement was seen in model 1, while the lowest displacement was seen in model 2 followed by model 4 (Figures 11–13, Table 5).

### 3.5. Displacements on the Global *Z*-Axis (Vertical, Intrusive-Extrusive)

#### 3.5.1. Model 1

**Figure 14:**
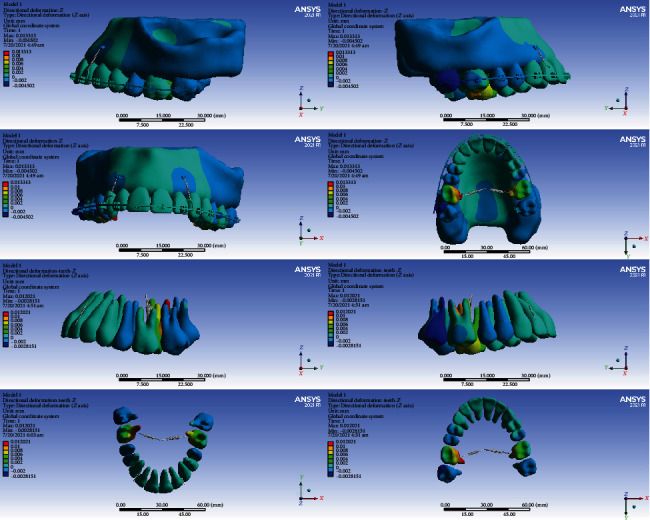
Displacements in the *Z* global direction (vertical) in model 1. Positive values indicate intrusion while negative values indicate extrusion.

In this model, the intrusion was seen in all the teeth except the second molars, the second premolars, and the right first premolar. The second premolars, the right first premolar, and the second molars were extruded (maximum extrusion: 0.002 mm). The maximum average of extrusion was seen in the second molars. The highest amount of intrusion was seen in the first molars. The maximum intrusion occurred in the palatal cusps of the first molars (maximum intrusion: 0.012 mm) (Figures 5 and 14, Table 6).

#### 3.5.2. Model 2

**Figure 15:**
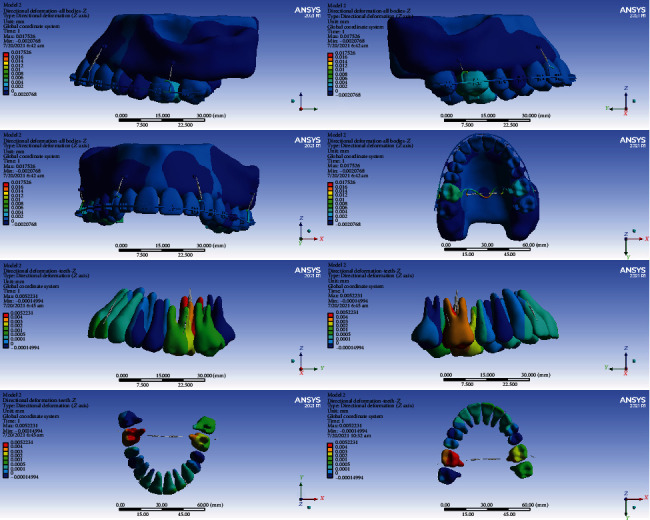
Displacements in the *Z* global direction (vertical) in model 2. Positive and negative values indicate intrusion and extrusion, respectively.

The left second premolar and the second right molar were extruded, but the other teeth were intruded. The maximum amount of intrusion in the anterior teeth was 0.0005 mm. The first molar teeth were intruded more than other teeth, and the amount of intrusion was higher at the palatal surfaces (maximum intrusion: 0.0052 mm) (Figures 5 and 15, Table 6).

#### 3.5.3. Model 3

**Figure 16:**
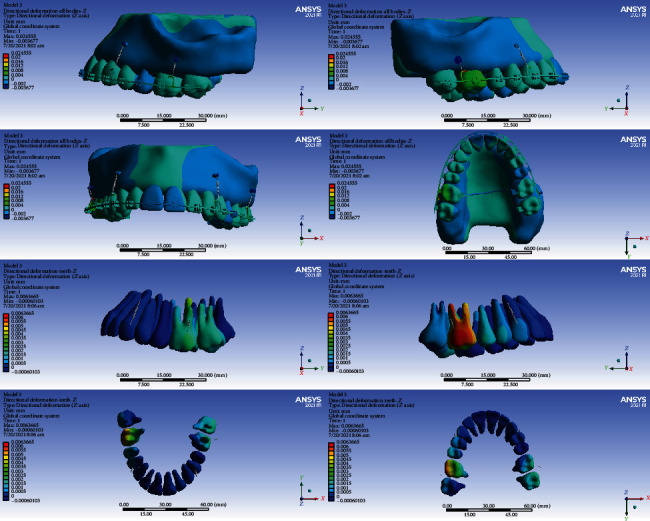
Displacements in the *Z* global direction (vertical) in model 3. Positive values indicate intrusion while negative values indicate extrusion.

The anterior teeth were intruded (maximum intrusion: 0.0005 mm). The highest amount of intrusion was observed in the right first molar (maximum displacement: 0.0052 mm). The second left molar showed the second maximum intrusion. Extrusion occurred in the right central and left second premolar (Figures 5 and 16, Table 6).

#### 3.5.4. Model 4

**Figure 17:**
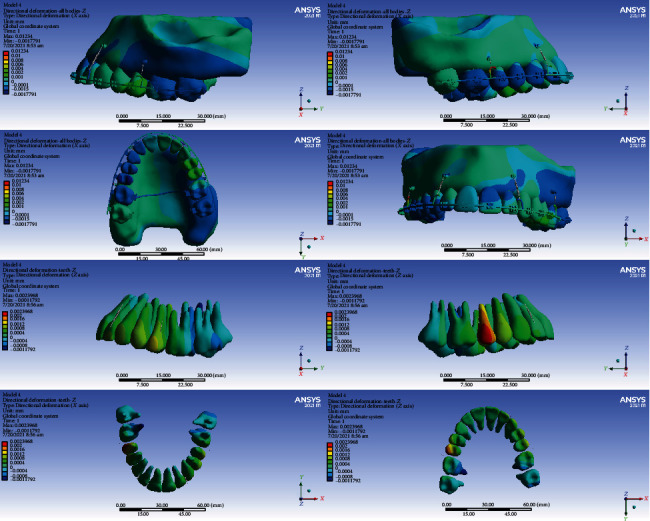
Displacements in the *Z* global direction (vertical) in model 4. Positive and negative values indicate intrusion and extrusion, respectively.

In this model, all teeth except molars were intruded. The highest extent of intrusion was seen in the premolars, especially the second premolar (maximum intrusion: 0.0024 mm). Palatal roots and palatal cusps of the molars were extruded (maximum extrusion: 0.0011 mm, Figures 5 and 17, Table6).

In the vertical dimension, the highest amounts of intrusion were seen in model 1 followed by model 2. The least intrusion occurred in the fourth model (Figures 14–17, Table 6).

The maximum and average extents of movement of each tooth in each model in the local directions of mesial, distal, buccal, lingual, intrusive, and extrusive have been illustrated in Figure 5.

### 3.6. All Body Stresses

#### 3.6.1. Model 1

**Figure 18:**
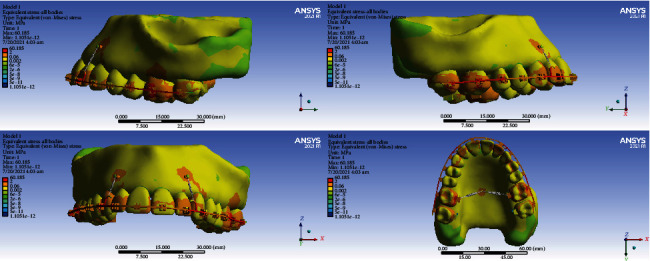
All body stresses (MPa) in model 1.

The highest level of stress was seen in the archwire between the lateral and canine teeth and between the second premolars, first molars, and second molars. High stresses were also seen in the lingual sheets of the first molar bands. The first and second molars, laterals, and canines tolerated the most stress among all teeth. A similarly high stress was observed in the bone around the miniscrew. Premolars received the least amount of stress (Figure 18).

#### 3.6.2. Model 2

**Figure 19:**
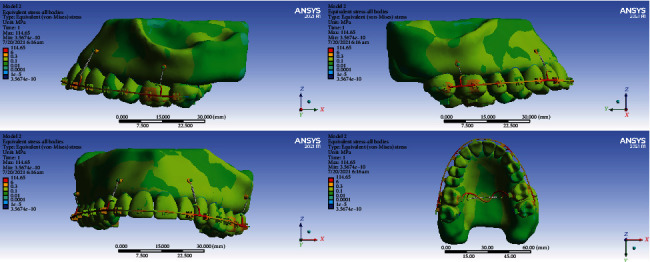
All body stresses (MPa) in model 2.

The highest stress was seen in the archwires in the area between the canine and lateral and between the first and second molars and the transpalatal arch. Less stress was exerted to the teeth. Small parts of the first and second molars were subjected to higher stress than other teeth (Figure 19).

#### 3.6.3. Model 3

**Figure 20:**
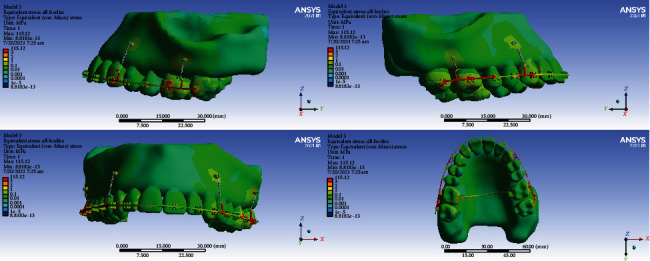
All body stresses (MPa) in model 3.

The highest amount of stress was seen mostly in the archwire between the second molar, first molar, and second molar and also between the canine and first premolar. Most teeth received similar stress levels except some parts of molars and canines that endured greater stresses (Figure 20).

#### 3.6.4. Model 4

**Figure 21:**
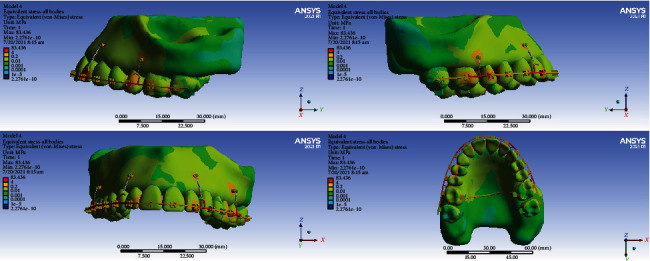
All body stresses (MPa) in model 4.

The highest stress extents were exerted to the archwire between the lateral and the first molar. The second premolars and first molars suffered the most amounts of stress (Figure 21).

## 4. Discussion

TADs (temporary anchorage devices) have increased orthodontic treatment capabilities by providing the desired movement of teeth in three dimensions, with their bone support. TADs are used in molar control, incisor segment control, molar distalization, and total arch displacement [19]. TADs are also used to treat skeletal problems. In patients with vertical maxillary excess who have excessive alveolar or gingival display, total arch intrusion is used [20–24]. In a recent review study, the use of miniscrews to reduce gingival appearance and improve gingival smile has been described as effective and practical [25]. The treatment of gingival hyperplasia using a miniscrew, with or without increasing the length of the periodontal crown, has advantages over orthognathic surgery such as lower risks, easier orthodontic biomechanics, less patient discomfort, increased cost-effectiveness, and not increasing the width of the alar base [26]. One of the main uses of TADs is the intrusion of the anterior teeth in patients with gummy smile: One of the main challenges of orthodontic treatment is the deep overbite correction. In most cases, this correction is caused by extrusion of the posterior teeth or a combination of anterior intrusion with posterior extrusion, which is undesirable in patients with vertical growth. In such cases, absolute anterior intrusion is necessary, especially when there is excessive incisors with extruded teeth. In particular, in cases where orthodontic opening of the posterior teeth using a bite plate or cervical retainer is contraindicated or unsuccessful, deep bite correction is possible only with the intrusion of the anterior teeth. In order to improve esthetics, patients with class 2 malocclusion with increased overjet and short-face height (who show increased gingival exposure of the incisor teeth at rest of the lips) are considered suitable candidates for such intrusion [27].

Comparing the first and second models, it was observed that in the second model, two buccal mini-implants were added in the distal region of the first molars, as well as a TPA. The results show that the addition of buccal force in the molar region does not increase the amount of molar intrusion, and that the molar and incisor intrusions remain higher in the first model. But the side effects of intrusion are reduced in the second model such that the rotation of the molars towards the palatal is reduced and the general mesial movement of the maxillary teeth compared to the first model is well controlled. Additionally, the labial movement of the incisors in the anterior region is also inhibited. Interestingly, despite the similarity of the anterior settings of the two models (#1 and #2), with the addition of posterior miniscrews, the amount of anterior intrusion decreased (without an increase in posterior intrusion). This decrease in anterior intrusion is probably a reaction to the increase in intrusive force at the posterior end of the wire. Comparing the third and fourth models, In model 4, the TADs are placed more mesially than in model 3 (in the third model, the implants are placed in the distal of the canine and molar, while in the fourth model, they are located in the mesial of these teeth). Therefore, in the fourth model, a greater mesial movement is observed in the posterior teeth. The placement of the posterior miniscrews in the mesial of the first molars drastically reduces the amount of molar intrusion. Whereas, in the incisor region, there is no clear difference in the extent of intrusion between the two models. Instead, the more mesial position of the posterior mini-implants in the third model has caused the highest amount of premolar intrusion in this model, in a way that unlike other models (which show the highest amount of dental arch intrusion in the molar area), in the third model, premolars are intruded more than any other tooth.

In this study, the palatal miniscrew in the first model (where a miniscrew was placed in the midpalatal between the first molars and two buccal miniscrews were placed between the laterals and canines) suffered the most stress. Buccal miniscrews in model 2 (which included a miniscrew in the midpalatal space between the first molars and four buccal miniscrews between the laterals and canines and between the molars) were less stressed than the other models. Therefore, it seems that in cases where the failure chance of the miniscrew is likely to be higher (due to the presence of patient-related factors such as younger age or poor oral hygiene [28]), the use of the second model is more useful. On the other hand, where it is necessary to use a smaller number of miniscrews and the stress on the miniscrew is not important, the first model that uses the least number of miniscrews is recommended. In the study of Gracco et al., the maximum stress was seen in the miniscrew head [29]. However, in the study of Fattahi et al. [30], the maximum stress was recorded in the lower parts of the miniscrew neck, which is in line with the present study. In another study [31], the pattern of stress distribution in miniscrews subjected to tooth intrusion was similar to our study. In the present study, the posterior miniscrews suffered more stress, which could be due to greater forces applied to them. The greatest stress among the models has been applied to the palatal miniscrew of the first model. By adding two buccal miniscrews in the second model, the stress of the palatal miniscrews has been reduced by almost half. And in this sense, it can be helpful in increasing the stability of palatal miniscrews.

Intrusion is a movement that makes the tooth prone to root resorption [32, 33]. If hydrostatic pressure exerted on the periodontal ligament is greater than the capillary pressure in the area, blood flow to that area will be impaired. Capillary pressure in the periodontal ligament is estimated to be around 0.002 to 0.005 MPa [11]. Based on the 0.0047-MPa threshold for compressive hydrostatic pressure as a risk factor for root resorption [10, 11], the followings were found to be areas prone to resorption: in model 1, the apical parts of the palatal root of the first molars; in model 2, the cervicobuccal area, the furca, and the palatal root of the first molars; in model 3, the cervicobuccal area of the second molars and the apical areas of the buccal roots of the first molars; and in model 4, the cervicobuccal and apical areas of the second molars and the apical areas and the cervicobuccal and apical areas of the buccal roots of first molars. In general, it seems that model 1 causes the highest compressive hydrostatic pressure in the periodontal ligament while model 4 causes the least stress, making it the most conservative one. According to Pizzo et al. [34], root resorption is an inflammatory process that leads to local ischemia of the periodontal ligament after applying force and is one of the most common complications of orthodontic treatment. The risk factors related to this complication include treatment-related factors such as the initial overjet size, amount of force, the direction of dental movement, and the method of applying force, treatment duration, and factors related to the patient, such as a person's sex, genetic predisposition, some systemic diseases, anomalies in root morphology, and dental trauma [34, 35]. Maxillary teeth may be more prone to root resorption than mandibular ones [36–39]. Among the maxillary teeth, the incisors are most prone to root resorption [37, 40]. In the maxillary arch, after the incisors, the molars are the next most prone to root resorption [38, 41]. In some studies, it has been stated that root resorption in premolars and molars may be trivial [36–38, 42]. In some studies, it has been reported that the intrusion movement has a great role in root resorption [33, 43–45]. This can be partially explained by the stress endured by the apex during intrusion [33, 46]. On the other hand, some studies did not show a relationship between intrusion and root resorption [33, 43, 47]. In a meta-analysis, it was asserted that the root resorption that occurs during intrusion is clinically within an acceptable range [33]. In the present study, the greatest risk for root resorption was seen in the first molars. In the fourth model, in addition to the first molars, the risk of root resorption was also seen in the second premolars. A higher amount of intrusion was seen in these teeth, which may be an associated with a greater probability of root resorption in these teeth. In an earlier study, the posterior intrusion was examined through the fine element method; they as well found the first molars to be susceptible to root resorption [48]. In our study, in addition to the apex of the molars, the furca area was also susceptible to root resorption. This was in line with the results of another study finding the furca as the most susceptible area to resorption during intrusion [49].

In our first model, the crowns of the anterior teeth were displaced buccally while their roots moved palatally. If the buccal tipping movement is indicated, for example in class II div 2 patients, this method can be useful in correcting dental inclination [13, 19]. In other models, the anterior teeth became palatalized, which can be helpful in patients with dental protrusion or class II div 1 patients [13, 19]. In the first and second models, the posterior teeth were palatalized; thus, in cases where the teeth have a buccal inclination, the use of these models is preferable [13, 19]. On the other hand, in the third and fourth models, the posterior teeth became buccalized, so in cases where the palatal inclination of the posterior teeth is desired, this model can be used [13, 19].

In our first model, the crowns of the premolars were mesialized, the crowns and roots of the second molars were distalized, and the palatal portions of the first molars were displaced to the mesial and their buccal portions to the distal. In the second model, the crowns of the first and second molars were distalized. In the third model, the premolars were distalized, the roots of the first molars were mesialized, and their crowns, especially their palatal cusps, were distalized; and the crowns of the second molars were mesialized and their roots distalized. In the fourth model, the crown of posterior teeth became mesialized and their roots were distalized.

In the vertical dimension, the highest amount of intrusion was seen in our first model followed by the second one. Therefore, when the amount of intrusion is crucial, it seems more practical to use these two methods. The least amount of intrusion occurred in the fourth model. The maximum intrusion of the premolars was seen in the fourth model; hence, this method may be preferred when it is important to control the movements of the premolars. The maximum intrusion of the first molas was observed in the first model, whereas, in the fourth model, the first molars were extruded. In the second molars, the maximum intrusion occurred in the third model; but in the first and fourth models, extrusion was observed (more in the first model). Such extrusions might be due to wire deflection. Overall, the fourth model does not seem to be successful in controlling the molar region. Few methods have been proposed in a few case reports for maxillary full-arch intrusion. However, the exact biomechanics of these methods, including the side effects of each of them on the anterior-posterior movement of the maxillary teeth (which can change the interarch relationship) or the resulting transverse dimensional changes, have not been systematically studied. Also in full-arch intrusion, the rotation of the occlusal plane during intrusion is very important. In patients with anterior open bite, a slight clockwise rotation during intrusion is desirable. While in patients with a long face with gummy smile, uniform intrusion in the anterior and posterior dental arch is preferred. Finite element analysis allows us to comprehensively evaluate the stress distribution and displacement of teeth in all three spatial dimensions for each model. In this study, we analyzed 4 models that at first glance seemed to effectively lead to uniform anterior and posterior maxillary intrusion, with minimal unwanted tooth movements in the anterior-posterior or transverse dimensions. However, this was not necessarily the case. Examination of our results shows that the first and second models cause a brief palatal movement of the crown of the posterior teeth, whereas, in the third and fourth models, these teeth move towards the buccal type. Therefore, the first two models should be used with caution in cases where there is a tendency for posterior crossbite before treatment or the roots of the posterior teeth are close to the buccal cortex. In the first and second models, where the posterior crowns became palatalized, the intrusive force was also applied from the palatal, but in the third and fourth models, despite the use of TPA, the teeth became buccalized. In the study of Kawamura et al. [50], it was reported that during posterior intrusion, buccal tipping of teeth occurred through buccal miniscrews, which recommended the use of TPA, which is stiffer, lingual constriction bend and lingual crown torque [50]. Only in the first model, the anterior teeth moved buccally, but in the other models, the anterior teeth became palatalized. The addition of two buccal miniscrews in the posterior side of the second, third, and fourth models may be effective in this regard. It is better not to use model 1 in cases where the anterior teeth are already in the buccal position and should not become more buccalized. In the first and second models (in which in addition to buccal miniscrews, there were also palatal miniscrews), the highest amount of intrusion was seen; it can be concluded that in total intrusion, the use of palatal miniscrews contributes to more effective intrusion. In the study of Till et al. [51] (in which the degree of distalization was examined), by placing two miniscrews between the premolars in addition to the interradicular miniscrews (located between the second premolar and the first molar), the molar distallization and retraction and incisor intrusion were considerably higher than the group that included only interradicular miniscrews between the second premolar and the first molar [51]. Between the first and second models, the second model, which used the greatest number of miniscrews, intruded more teeth. In the fourth model, besides the lowest amount of intrusion that was observed, the vertical control on the molars was minimal and the highest amount of intrusion was in the premolar area, which can be justified by the more anterior position of the posterior miniscrews. This model is recommended in cases where the inclination of the occlusal plane is such that the need for intrusion in the premolars is the maximum. It seems that increasing the number of miniscrews has a positive effect on controlling the buccolingual dimension of the posterior teeth and the mesiodistal dimension of the anterior teeth, because, in the second model with the most miniscrews, the least dental displacements in the said dimensions were observed. On the other hand, the highest displacements were detected in the first model with the fewest miniscrews.

There is no FEA study on full-arch intrusion. Nevertheless, there are 3 finite element analyses examining intrusion of the anterior teeth. A 3D finite element model was created for six anterior teeth [52]. After adjusting the alveolar bone loss to 0, 2 or 4 mm, the positions of the miniscrews and hooks were changed. Then, the primary displacement of each tooth in three directions and the amount of labial tilt after applying 100 grams of intrusive force were measured. The findings showed that with the reduction of alveolar bone height, the amount of labial tilt increased under the same load. When a miniscrew was placed between two central teeth, the mediolateral and anterior-posterior displacements of the central incisor were significantly greater than other cases. In the case where the miniscrew was placed in the distal of canines (and the distal intrusion force was applied to the lateral incisors), the amount of labial tilting and displacement of the six anterior teeth was the lowest, and the maximum stress was uniformly distributed in all teeth [52]. In another study, intrusive loading of maxillary incisors was simulated [53]. The force application points were the following: the central area between the brackets of the central incisors, bilaterally between the brackets of the central and lateral incisors, at the distal of the brackets of the lateral incisors on both sides, and 7 mm distal to the center of the brackets of the lateral incisors on both sides. The results showed that the stress (regardless of the application point of the orthodontic force) was concentrated in the PDL of the root apex region. Four loading models showed different compressive stress values compared to the midsagittal reference line. Stress distributions in the central and lateral incisors were not the same in a similar loading model. When the force application point was in the distal of the brackets of the lateral incisors, a more balanced compressive stress distribution was seen [53]. In the third study, the finite element model was created from the central teeth to the maxillary first premolar [54]. Four different modes of intrusion mechanics were simulated with different placement locations for the miniscrews as well as different force application points. In each model, a force of 25 g was applied to the maxillary incisors. In all four models, there was an increase in stress values in the apical region of the lateral incisor. Proclination of maxillary incisors was also reported in all four models. The absolute minimum intrusion was observed when the miniscrew was placed between the lateral incisors and canines, and the force was applied at a right angle to the archwire (which is very common in clinical treatments). It seemed that the apical region of the lateral incisor was the most susceptible place for root resorption during the intrusion of anterior teeth. In clinical situations where minimum flaring of the maxillary incisors is required, it is suggested that miniscrews be placed between the roots of the lateral incisors and canines, and the force be applied between the central and lateral incisors. In order to achieve maximum absolute intrusion, it is recommended to place the miniscrew between the roots of the central and lateral incisors and apply the force at a right angle to the archwire between the two teeth [54].

This in silico simulation was limited by some factors. A limitation for finite element modeling is its theoretical approach. Based on the hypotheses derived from the average properties of bone or teeth or other structures, this analysis is basically a static analysis that is difficult to apply in clinical conditions; thus, careful decisions must be made to realize its modeling and analysis [55]. Finite element studies only examine very short-lived and very fast and also static *mechanical* relationships. They do not and cannot examine the *biological* changes happening over a long period of time [56]. Root resorption has multiple potential mechanisms including the engagement of the root with the cortical bone. However, the movement of the roots towards the bone cortex and its engagement with the cortical bone happens over a long period of time and has biological mechanisms. Therefore, it is impossible for finite element studies to examine such dynamics which occur in long term and through biological mechanisms. Similarly, finite element studies cannot examine the biological changes happening in a very long time needed for the establishment of the secondary stability of miniscrews. However, since clinical examination of novel and unknown methods are not ethical in many situations, FEA simulations can act as a beginning point in a chain of research to be followed by later animal and clinical studies [56]. Therefore, future animal and clinical studies are needed to verify our results. Furthermore, future research should also examine zygomatic miniplates. They also should include in the “Mouse Trap” model, which is among the most widespread treatments for the solution of this clinical problem. The application of TAD in the palatal area at the height of the third palatine wrinkle can present significant biomechanical and biological advantages in this approach.

## 5. Conclusions

The following conclusions can be summarized: (1) The highest amounts of incisor and molar intrusion were seen in the first model. With the addition of buccal alveolar miniscrews in the posterior region in the second model (along with the TPA placement), the extents of incisor and molar intrusion were reduced compared to the first model, and at the same time, unwanted movements in other planes (such as palatal and mesial movements of molars and labial movements of incisors) were inhibited. (2) The more-mesial placement of the posterior miniscrews in the fourth model (in the mesial of the first molars) severely reduced the intrusion of the molars and instead increased the intrusion in the premolar area, so that the fourth model showed the highest premolar intrusion compared to other models. (3) In the first three models, the highest amount of intrusion occurred in the first molar region; in the fourth model, it was seen in the premolar area. The overall amounts of intrusion were highest in the first model followed by the second one; therefore, it seems that these might be more practical when a greater extent of intrusion is needed. (4) In general, it can be concluded that in model 1, compared to other models, the highest compressive hydrostatic stress is seen in the periodontal ligament, while in model 4, the least compressive stress is seen. Hence, it seems that the use of the fourth model is more conservative.

## Figures and Tables

**Table 1:** Material properties.

Material	Elastic modulus (MPa)	Poisson ratio
Cortical bone [14]	1000	0.3
Cancellous bone [14]	500	0.3
Dentine [14]	18600	0.3
PDL [15]	0.15	0.45
Stainless steel [16]	200000	0.3
Miniscrew titanium G5 [17]	115000	0.33

**Table 2:** Stresses of miniscrews (MPa).

Model	Miniscrew	Scope	Min.	Max.	Avg.
1	Palatal	Whole body	0.0000008	16.1210000	1.4865000
		Thread	0.0000008	16.1210000	1.9217000
	Left	Whole body	0.0001052	4.1372000	0.7149800
		Thread	0.0282100	4.1372000	0.7925700
	Right	Whole body	0.0001071	3.9836000	0.7328300
		Thread	0.0068716	3.9836000	0.8668500

2	Palatal	Whole body	0.0000524	7.2858000	0.6973300
		Thread	0.0000524	7.2858000	0.9712000
	Posterior-left	Whole body	0.0001009	5.8670000	0.8523300
		Thread	0.0527440	5.8670000	0.9218400
	Anterior-right	Whole body	0.0003064	4.4316000	0.7194800
		Thread	0.0194830	4.4316000	0.7812000
	Anterior-left	Whole body	0.0002860	4.4129000	0.7308100
		Thread	0.0095590	4.4129000	0.7899000
	Posterior-right	Whole body	0.0001355	5.9749000	0.8340100
		Thread	0.0100770	5.9749000	0.9612800

3	Posterior-left	Whole body	0.0001892	10.7130000	1.6071000
		Thread	0.1332100	10.7130000	1.7457000
	Anterior-right	Whole body	0.0000396	5.6620000	0.9710100
		Thread	0.0112180	5.6620000	1.0831000
	Anterior-left	Whole body	0.0000861	8.2548000	1.3595000
		Thread	0.0356900	8.2548000	1.6299000
	Posterior-right	Whole body	0.0002547	10.8870000	1.5293000
		Thread	0.0269770	10.8870000	1.7300000

4	Posterior-left	Whole body	0.0006735	10.5660000	1.5098000
		Thread	0.0356050	10.5660000	1.7541000
	Anterior-right	Whole body	0.0003839	5.5155000	0.8957400
		Thread	0.0321420	5.5155000	0.9818700
	Anterior-left	Whole body	0.0003572	5.5292000	0.9039300
		Thread	0.0199810	5.5292000	0.9948500
	Posterior-right	Whole body	0.0002007	13.9100000	1.8658000
		Thread	0.0229710	13.9100000	2.2215000

Min: minimum; Max: maximum; Avg: average.

**Table 3:** Hydrostatic stresses of the PDL (kilopascal (kPa)). Positive values indicate tensile stresses while negative values show compressive pressures. Negative values below -4.7 kPa (i.e., compressive pressures above 0.0047 MPa) pose a considerably higher external root resorption risk.

*N*	Tooth	Model 1			Model 2			Model 3			Model 4		
		Min.	Max.	Avg.	Min.	Max.	Avg.	Min.	Max.	Avg.	Min.	Max.	Avg.
2	Right second molar	-3.071	3.965	0.635	-1.875	0.922	-0.282	-4.976	2.621	-0.692	-0.868	0.472	-0.105
3	Right first molar	-20.021	11.591	-2.794	-10.937	5.149	-2.378	-15.184	7.135	-2.154	-3.05	2.41	-0.04
4	Right second premolar	-0.769	0.996	0.043	-0.858	0.38	-0.174	-2.69	1.277	-0.657	-5.014	2.832	-0.829
5	Right first premolar	-0.459	0.22	0.003	-0.465	0.151	-0.067	-1.67	0.724	-0.331	-1.737	0.607	-0.248
6	Right canine	-0.926	0.576	-0.139	-0.76	0.663	-0.122	-2.285	0.533	-0.235	-1.21	0.72	-0.172
7	Right lateral	-1.046	0.524	-0.142	-1.066	0.432	-0.111	-0.84	0.593	-0.033	-1.49	0.826	-0.126
8	Right central	-0.913	0.687	-0.201	-0.91	0.372	-0.149	-0.613	0.607	0.007	-1.073	0.273	-0.158
9	Left central	-1.466	0.991	-0.237	-1.03	0.638	-0.187	-0.588	0.498	-0.049	-1.408	1.212	-0.223
10	Left lateral	-2.05	1.279	-0.217	-1.554	0.885	-0.16	-0.766	0.598	-0.047	-2.046	1.673	-0.188
11	Left canine	-1.589	0.415	-0.27	-1.288	0.357	-0.204	-1.811	0.741	-0.26	-2.173	1.09	-0.29
12	Left first premolar	-0.765	0.436	-0.097	-0.352	0.18	-0.069	-1.638	0.92	-0.151	-1.711	1.645	-0.117
13	Left second premolar	-0.492	0.428	-0.001	-0.284	0.121	-0.022	-0.463	0.148	-0.054	-4.752	3.721	-0.2
14	Left first molar	-16.417	7.337	-2.846	-6.945	3.349	-1.638	-6.16	3.527	-0.625	-3.207	2.113	-0.202
15	Left second molar	-1.984	1.832	-0.1	-2.811	0.928	-0.654	-5.56	2.691	-1.124	-0.932	0.779	-0.08

*N*: tooth number based on the Universal Dental Notation system; Min: minimum; Max: maximum; Avg: average.

**Table 4:** Displacements (*μ*m) in the global *Y*-axis (anterior-posterior). Positive values indicate posterior movement while negative values indicate anterior movement.

*N*	Tooth	Model 1			Model 2			Model 3			Model 4		
		Min.	Max.	Avg.	Min.	Max.	Avg.	Min.	Max.	Avg.	Min.	Max.	Avg.
2	Right second molar	-0.8561	1.6522	0.1938	-0.006	0.2233	0.085	-0.2819	0.1539	-0.0182	-0.2702	0.0491	-0.0825
3	Right first molar	-4.4451	3.6742	-0.0588	0.2849	0.94	0.5937	-0.3746	1.6572	0.5277	-0.7248	0.3685	-0.1214
4	Right second premolar	-0.4747	0.0126	-0.1977	-0.205	0.309	0.0653	-0.0903	0.4152	0.1971	-1.0682	0.4264	-0.2206
5	Right first premolar	-0.5514	0.0474	-0.2339	-0.0109	0.1846	0.0715	0.1904	0.2988	0.2404	-0.2016	0.2211	0.0305
6	Right canine	-0.5068	0.1192	-0.1744	-0.0133	0.2091	0.1092	0.1429	0.2944	0.2377	-0.0388	0.3484	0.1678
7	Right lateral	-0.6167	0.2064	-0.1417	-0.0709	0.3835	0.1894	0.1772	0.3291	0.2366	0.0551	0.612	0.3853
8	Right central	-0.7524	0.1524	-0.2853	-0.1219	0.2207	0.0678	0.0292	0.3392	0.1873	0.2208	0.3529	0.3163
9	Left central	-0.7967	0.2976	-0.3204	-0.1778	0.3577	0.0599	-0.0064	0.2915	0.1368	-0.0559	0.6699	0.3281
10	Left lateral	-0.7897	0.4918	-0.1663	-0.2357	0.5366	0.1476	0.0254	0.138	0.0788	-0.4363	0.9696	0.3074
11	Left canine	-0.7273	0.3051	-0.1662	-0.19	0.2689	0.0788	-0.0702	0.2477	0.1039	-0.6953	0.388	-0.0591
12	Left first premolar	-0.7138	0.0996	-0.3635	-0.161	0.1233	-0.0206	-0.1526	0.2661	0.0419	-0.9571	0.1787	-0.4609
13	Left second premolar	-0.6716	-0.0189	-0.2829	-0.1153	0.0619	-0.0012	-0.117	0.0747	0.0094	-1.1324	0.1277	-0.4888
14	Left first molar	-1.3722	1.465	0.1824	-0.9224	1.2803	0.1117	-0.885	1.0071	-0.0743	-1.6125	0.9381	-0.1241
15	Left second molar	0.0343	0.4685	0.2286	-0.004	0.3259	0.1628	-0.6031	0.4833	0.0001	-0.7531	0.1258	-0.2307

*N*: tooth number based on the Universal Dental Notation system; Min: minimum; Max: maximum; Avg: average.

**Table 5:** Displacements (*μ*m) in the global *X* axis (left-right). Positive values indicate displacements to the patient's left side while negative values indicate movements to the patient's right side.

*N*	Tooth	Model 1			Model 2			Model 3			Model 4		
		Min.	Max.	Avg.	Min.	Max.	Avg.	Min.	Max.	Avg.	Min.	Max.	Avg.
2	Right second molar	0.2216	3.4492	1.5025	0.15	0.4423	0.3237	-1.1963	0.8465	0.0108	0.0213	0.0939	0.0665
3	Right first molar	-4.4648	15.394	4.1755	0.6511	2.8208	1.585	-4.663	3.6657	0.1074	-0.9887	0.8839	0.1123
4	Right second premolar	0.3454	1.1381	0.7602	0.2028	0.4756	0.3382	-1.0895	1.0665	-0.2038	-1.7791	1.8821	-0.2537
5	Right first premolar	-0.0276	0.8414	0.4455	0.0832	0.2457	0.1504	-0.9842	0.9262	-0.2239	-1.1659	0.7048	-0.3645
6	Right canine	-0.0383	0.4762	0.1835	-0.0103	0.1161	0.0649	-0.7483	0.4497	-0.1082	-0.6504	0.4443	-0.0798
7	Right lateral	-0.0088	0.1921	0.1074	-0.08	0.1234	0.0391	-0.4353	0.0502	-0.1622	-0.279	0.2373	0.0282
8	Right central	-0.0831	0.0601	-0.0169	-0.0876	0.0127	-0.0439	-0.3611	-0.0764	-0.2397	-0.1023	0.0357	-0.0276
9	Left central	-0.3474	0.0597	-0.1256	-0.265	0.0096	-0.1182	-0.3741	-0.2147	-0.2896	-0.3244	0.1549	-0.0816
10	Left lateral	-0.4709	0.0706	-0.2204	-0.3466	0.0436	-0.1652	-0.4233	-0.1486	-0.2968	-0.496	0.3966	-0.0569
11	Left canine	-0.3997	-0.0361	-0.1876	-0.2218	-0.0204	-0.102	-0.3367	-0.0542	-0.2432	-0.212	0.805	0.218
12	Left first premolar	-0.6432	-0.0597	-0.3962	-0.3566	-0.0239	-0.2147	-0.199	-0.1038	-0.1569	-0.4772	1.3827	0.6526
13	Left second premolar	-0.8667	-0.1302	-0.4824	-0.4893	-0.0817	-0.2765	-0.2604	-0.1429	-0.2032	-0.3755	1.9971	0.7191
14	Left first molar	-10.66	2.3408	-3.2119	-2.8718	-0.229	-1.3595	-2.1271	2.1591	-0.3178	-0.6387	0.0659	-0.3545
15	Left second molar	-2.2129	-0.9151	-1.435	-0.6705	-0.153	-0.4767	-0.626	1.0607	0.0296	-0.5451	-0.2265	-0.3542

*N*: tooth number based on the Universal Dental Notation system; Min: minimum; Max: maximum; Avg: average.

**Table 6:** Movements (*μ*m) in the global *Z*-axis (vertical, intrusive-extrusive). Positive values indicate intrusion while negative values indicate extrusion.

*N*	Tooth	Model 1			Model 2			Model 3			Model 4		
		Min.	Max.	Avg.	Min.	Max.	Avg.	Min.	Max.	Avg.	Min.	Max.	Avg.
2	Right second molar	-2.8151	0.0506	-1.4054	-0.1441	0.0638	-0.0647	-0.4464	0.9838	0.2346	-0.144	0.045	-0.0406
3	Right first molar	-1.1411	12.021	4.9139	3.5069	5.2231	4.283	0.2378	6.3665	3.6323	-1.1792	0.5967	-0.2499
4	Right second premolar	-0.5503	-0.0816	-0.2979	-0.011	0.1612	0.0766	0.2935	1.5269	0.8597	0.1987	2.3968	1.194
5	Right first premolar	-0.33	0.2424	-0.0198	-0.037	0.0497	0.0083	0.0107	1.0739	0.4913	-0.0636	1.1668	0.4724
6	Right canine	0.0664	0.5563	0.2918	0.0027	0.1988	0.0716	-0.0052	0.6591	0.2708	-0.1004	0.5573	0.2096
7	Right lateral	0.2236	0.9793	0.5303	-0.0596	0.3874	0.1351	-0.0172	0.2807	0.0796	-0.2476	0.4227	0.0484
8	Right central	0.4613	1.2538	0.8519	0.1715	0.4795	0.3102	-0.1311	0.1132	-0.0155	0.0652	0.2123	0.121
9	Left central	0.4476	1.3839	0.9419	0.1172	0.6051	0.3602	-0.1265	0.1143	0.0053	-0.1322	0.5661	0.1665
10	Left lateral	0.2897	1.3758	0.8061	-0.0127	0.6568	0.3034	0.0331	0.2269	0.1021	-0.3785	0.878	0.2107
11	Left canine	0.2745	1.0475	0.5943	0.1167	0.4656	0.2268	0.0341	0.3423	0.1519	-0.0428	0.888	0.3876
12	Left first premolar	0.0688	0.4701	0.2586	-0.0436	0.1596	0.0586	-0.0336	0.2173	0.0637	-0.103	1.0834	0.4485
13	Left second premolar	-0.2358	0.1752	-0.0433	-0.1499	0.0495	-0.0497	-0.1214	-0.0106	-0.0775	-0.2225	1.3321	0.455
14	Left first molar	0.373	8.8284	4.0164	1.2881	3.712	2.4076	-0.601	2.2099	0.8569	-0.7636	0.8948	-0.0348
15	Left second molar	-0.8398	0.167	-0.3456	0.4423	0.7901	0.6189	0.654	1.9433	1.2819	-0.5082	-0.0085	-0.1852

*N*: tooth number based on the Universal Dental Notation system; Min: minimum; Max: maximum; Avg: average.

## Data Availability

All data are presented as figures and tables.
